# Supplementary material for: Development and validation of a portable, point-of-care canine distemper virus qPCR test
Source: PLoS One. 2020 Apr 22;15(4):e0232044. doi: 10.1371/journal.pone.0232044 (PMC7176111; doi:10.1371/journal.pone.0232044)
Supplement: S1 Table — qPCR results from 26 samples of CDV suspect animals comparing standard laboratory practices at the Virology Laboratory (University of Veterinary Medicine, Vienna, Austria) to the Biomeme POC platform. (DOCX) [file pone.0232044.s001.docx]

**S1 Table.**

| **Animal** | **ID** | **Species** | **Common name** | **Organ** | **Biomeme POS Platform Results** | **Independent Virology lab Results** |
| --- | --- | --- | --- | --- | --- | --- |
| **1** | **AC 569/18** | ***V. vulpes*** | **Red fox** | **liver** | **POS** | **POS** |
|  | **AC 569/18** | ***V. vulpes*** | **Red fox** | **kidney** | **POS** | **POS** |
| **2** | **AC 570/18** | ***V. vulpes*** | **Red fox** | **liver** | **POS** | **POS** |
|  | **AC 570/18** | ***V. vulpes*** | **Red fox** | **kidney** | **POS** | **POS** |
| **3** | **AC 571/18** | ***V. vulpes*** | **Red fox** | **kidney** | **NEG** | **NEG** |
| **4** | **2054** | ***L. lutra*** | **Otter** | **lung** | **NEG** | **NEG** |
|  | **2054** | ***L. lutra*** | **Otter** | **brain** | **POS (NEG on retest)** | **NEG (NEG on retest)** |
|  | **2054** | ***L. lutra*** | **Otter** | **liver** | **NEG** | **NEG** |
|  | **2054** | ***L. lutra*** | **Otter** | **kidney** | **NEG** | **NEG** |
| **5** | **889** | ***M. meles*** | **Badger** | **heart** | **POS** | **POS** |
|  | **889** | ***M. meles*** | **Badger** | **kidney** | **POS** | **POS** |
|  | **889** | ***M. meles*** | **Badger** | **lung** | **POS** | **POS** |
|  | **889** | ***M. meles*** | **Badger** | **brain** | **POS** | **POS** |
| **6** | **2523** | ***M. meles*** | **Badger** | **kidney** | **POS** | **POS** |
|  | **2523** | ***M. meles*** | **Badger** | **lung** | **POS** | **POS** |
|  | **2523** | ***M. meles*** | **Badger** | **brain** | **POS** | **POS** |
| **7** | **2502** | ***M. meles*** | **Badger** | **lung** | **POS** | **POS** |
|  | **2502** | ***M. meles*** | **Badger** | **brain** | **POS** | **POS** |
| **8** | **AC 909/12** | ***M. foina*** | **Beech marten** | **kidney** | **POS** | **POS** |
|  | **AC 909/12** | ***M. foina*** | **Beech marten** | **lung** | **POS** | **POS** |
|  | **AC 909/12** | ***M. foina*** | **Beech marten** | **brain** | **POS** | **POS** |
| **9** | **AC 910/12** | ***M. martes*** | **Pine marten** | **kidney** | **POS** | **POS** |
|  | **AC 910/12** | ***M. martes*** | **Pine marten** | **lung** | **POS** | **POS** |
|  | **AC 910/12** | ***M. martes*** | **Pine marten** | **brain** | **POS** | **POS** |
| **10** | **6467** | ***V. vulpes*** | **Red fox** | **kidney** | **NEG** | **NEG** |
|  | **6467** | ***V. vulpes*** | **Red fox** | **lung** | **NEG** | **NEG** |
