# Supplementary material for: Development and validation of a portable, point-of-care canine distemper virus qPCR test
Source: PLoS One. 2020 Apr 22;15(4):e0232044. doi: 10.1371/journal.pone.0232044 (PMC7176111; doi:10.1371/journal.pone.0232044)
Supplement: Attachment — Submitted filename: POC_BioMeme_CVD.docx [file pone.0232044.s002.docx]

Overall, this is a well-written manuscript with sound methodology and the authors have clearly conveyed the importance of the development of a field-deployable point-of-care diagnostic assay for canine distemper virus. The results show that the establishment of this CDV Biomeme assay has the potential to be a useful diagnostic tool for both wildlife biologists and in conservation efforts. The confirmation of hair as a reliable sample source for CDV diagnostics is particularly advantageous as risk is minimized to field personnel without compromising the ability to generate rapid, actionable data.

I am concerned by the disagreement between the laboratory standard assay and the Biomeme assay. The authors report that one sample derived from brain tissue was reported as positive by the Biomeme assay, but negative with the standard assay. This is inconsistent with the frequent discussion point throughout the manuscript that the Biomeme assay is less sensitive than the laboratory standard assay. The manuscript would be improved by the inclusion of a follow-up to confirm the results for the sample in question with both assays.

I believe that the study and described assay is a valuable addition to the field in its current state. As the authors note, clinical manifestations of rabies virus in wildlife may be confused for any number of other pathogens including CDV; however, rabies virus poses a high risk to field personnel. The assay could be improved in the future with the development of a multiplexed assay targeting CDV, Rabies virus, and an internal control with low variability between related species (e.g. HPRT or GAPDH) to ensure that the nucleic extraction was successful. ­­
